# Targeting the latent reservoir to achieve functional HIV cure

**DOI:** 10.12688/f1000research.8109.1

**Published:** 2016-05-26

**Authors:** Daniele C. Cary, B. Matija Peterlin

**Affiliations:** 1 Departments of Medicine, Microbiology and Immunology, University of California at San Francisco, San Francisco, CA, USA

**Keywords:** HIV, latent HIV, targeting latent HIV, viral reactivation, host defense mechanisms, gene therapy

## Abstract

While highly active anti-retroviral therapy has greatly improved the lives of HIV-infected individuals, current treatments are unable to completely eradicate the virus. This is due to the presence of HIV latently infected cells which harbor transcriptionally silent HIV. Latent HIV does not replicate or produce viral proteins, thereby preventing efficient targeting by anti-retroviral drugs. Strategies to target the HIV latent reservoir include viral reactivation, enhancing host defense mechanisms, keeping latent HIV silent, and using gene therapy techniques to knock out or reactivate latent HIV. While research into each of these areas has yielded promising results, currently no one mechanism eradicates latent HIV. Instead, combinations of these approaches should be considered for a potential HIV functional cure.

## Introduction

In the twenty years since the implementation of highly active anti-retroviral therapy (HAART), the overall face of HIV as a global health issue has changed ^1^. HAART—composed of a cocktail of anti-retroviral drugs which target proteins expressed at different steps in the HIV replication cycle—can affect only cells that harbor actively replicating virus. HIV+ individuals are able to live fairly normal lives on maintenance HAART, with minimal side effects. Nevertheless, the effects of HIV infection continue to be evident in these suppressed individuals, who continue to suffer from a number of metabolic, immunologic, and neurologic co-morbidities ^2^. Thus, despite reducing plasma viremia below detection limits, the virus is not eliminated. There is evidence that low levels of replication occur in suppressed individuals, primarily in tissue reservoirs; however, this is not reflected in systemic plasma viremia in these individuals ^3, 4^. HAART requires life-long administration. Following even brief treatment interruption, HIV rebounds rapidly from its reservoirs ^5– 7^. Goals of the present research are to eliminate, suppress permanently, or render cells inhospitable to the hidden HIV in infected individuals.

**Figure 1.**
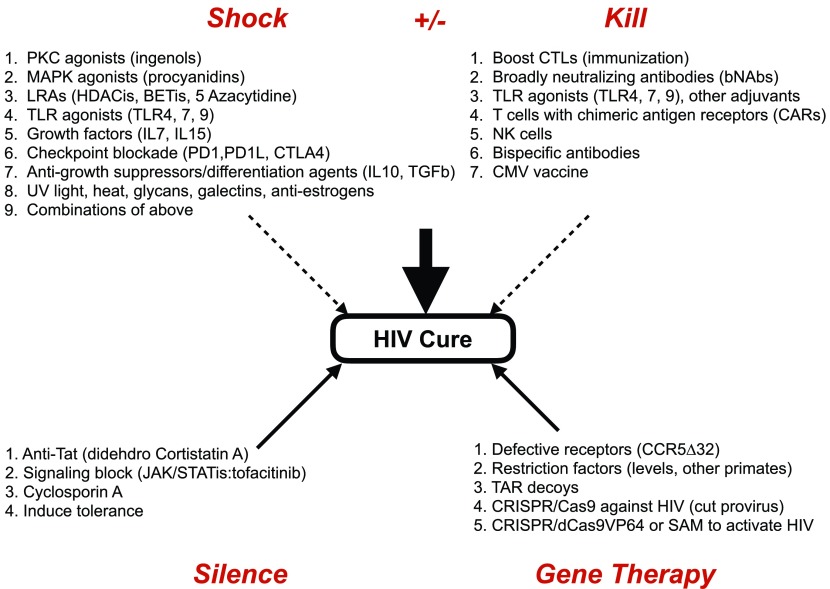
Four main approaches that target the latent reservoir of HIV Four research areas, which reactivate HIV (1. shock), eliminate HIV (2. kill), silence HIV (3. silence), or alter the immune system to resist HIV (4. gene therapy) should contribute to the functional or complete cure of HIV in infected individuals. Within each area are individual components of that therapy. They can be applied individually or in combinations, which should decrease their doses and deleterious effects. Most likely, there will be additional approaches in the future.

Research efforts to understand and target HIV reservoirs have focused on four main categories outlined in this review ( Figure 1): first, reactivation of latent HIV by capitalizing on the ability of host cellular activation signals and transcription factors (TFs) to ‘shock’ the virus out of hiding; second, killing of reactivated HIV by strengthening the immune system, which has been crippled by the infection; third, keeping latent reservoirs permanently suppressed; and, finally, targeting HIV and CD4+ T cells, which are the primary host cells for the virus, via new gene therapy approaches.

## Shock

Chronic infection by HIV is characterized by severe depletion of CD4+ T cells and continuing inflammation, which contribute to HIV-associated co-morbidities ^2^. Continued exposure to inflammatory cytokines exhausts the immune system. It also elevates the expression of the receptors programmed death 1 (PD-1) ^8^ and cytotoxic T-lymphocyte–associated antigen 4 (CTLA-4) ^9^ on T cells. Blockade of these molecules is used as a treatment for solid tumors ^10^ and could reinvigorate exhausted T cells in HIV+ patients ^11^. These individuals also produce elevated levels of inhibitory cytokines interleukin (IL)-10 and transforming growth factor–beta (TGF-β) ^12, 13^. Indeed, blocking IL-10 results in increased T cell activity in a hepatitis C infection model ^14, 15^.

Growth factor therapy, including treatment with IL-2, -7, or -15, is being explored as a means to stimulate T cell recovery. IL-2 and IL-7 are important T cell growth and proliferation factors. Infusion with IL-2 and IL-7 results in enhanced T cell production and memory T cell proliferation ^16– 18^. IL-15 enhances cytotoxic CD8+ T lymphocyte (CTL) and natural killer (NK) cell activity *in vitro*. Indeed, the IL-15 super-agonist ALT-803 is currently in preclinical trials ^19^.

Latent HIV is primarily found in resting CD4+ T cells in the periphery. Resting cells have low levels of cellular TFs, which are also required for HIV replication, including NF-κB, P-TEFb, and CDK11 ^20, 21^. Among the first examined latency reversing agents (LRAs) were histone deacetylase inhibitors (HDACis) and BET bromodomain inhibitors (BETis), which induce chromatin stress and induce the release of positive transcription elongation factor b (P-TEFb) from its repressive complex ^22^. HDACis—such as panobinostat ^23^, romidepsin ^24^, SAHA ^25^, and valproic acid ^26^—and BETis—such as JQ1 ^27^—all reactivate HIV in cell line models of latency. However, they do not work in human primary resting infected T cells ^28, 29^ because they contain very low levels of necessary TFs ^20, 21^. Thus, clinical trials with SAHA resulted in only a modest and transient reactivation of HIV ^30^, making it an impractical mono-therapy for HIV reactivation.

Since HDACis and BETis do not increase levels of required TFs, some activation of CD4+ T cells is required. Indeed, protein kinase C (PKC) agonists, such as prostratin ^31^ and bryostatin ^32^, and the MAPK agonist procyanidin ^33, 34^ can reactivate HIV in cell line models and primary CD4+ T cells. However, prostratin is toxic at therapeutic levels, leading to muscle pain, respiratory distress, and hypertension. Bryostatin, derived from a marine animal, *Bugula neritina*, not only has similar side effects but is also cost prohibitive to manufacture. Because of these limitations, a number of synthetic analogues of prostratin and bryostatin with reduced toxicity *in vitro* are being developed ^35– 37^. Ingenols, which are purified from Euphorbia plants, represent additional PKC agonists of interest. Native and chemically modified ingenols reactivate HIV in cell lines and primary T cells ^38– 40^. These PKC agonists also increase cellular levels of necessary TFs ^38^. Thus, select MAPK and PKC agonists represent attractive candidates to reactivate latent HIV.

Combining several of these approaches has the greatest potential to purge the viral reservoir. Indeed, lower doses of a T cell activator and an LRA (HDACi or BETi) can be administered for increased potency and reduced pro-inflammatory responses ^41– 43^. Further understanding of HIV integration, transcription, and reactivation, as well as host cell behaviors, will inform optimal combinations of activators and LRAs.

## Kill

Strategies to remove HIV by enhancing the killing by CTL and NK cells ^44^ or via broadly neutralizing antibodies (bNAbs) represent the second major field of research in HIV eradication. It is also important to investigate kill strategies in the context of the aforementioned shock therapies because many of the treatments proposed to reactivate latent HIV also dampen CTL function ^45^, which is already impaired in HIV+ individuals ^11^.

Using modified cytomegalovirus (CMV), a live vaccine expressing several simian immunodeficiency virus (SIV) antigens, was found to protect rhesus macaques against viral challenge ^46– 48^. Vaccinated animals initially appeared to be infected; however, they gained protection against SIV and showed enhanced effector T cell function against viral antigens.

Another approach involves bNAbs ^49^. Following infection, anti-HIV antibodies are abundant in HIV+ patients; however, owing to the ability of the virus to mutate, the majority of them fail to eliminate the virus. bNAbs are the exception, in that they recognize many clades of HIV as well as escape mutants of the virus. In several studies, they not only neutralized virions released from activated CD4+ T cells from patients ^50^ but also reduced the viral rebound following HIV reactivation in a humanized mouse model ^51^. However, even the most potent bNAbs are each only effective against a narrow subset of HIV clinical isolates, suggesting that effective bNAb approaches may require a combination of several bNAbs ^52^. A second antibody approach utilizes bispecific antibodies, wherein one arm of the Fab portion of the antibody recognizes HIV envelope and the second arm recognizes CD3, making the cell vulnerable to CTL-mediated killing.

Finally, in an effort to achieve more effective killing, chimeric antigen receptors (CARs), which increase T cell receptor avidity and activation, are being explored. They can be engineered to recognize specific viral proteins; CARs against CD19, which is a B cell receptor, led to an astounding 90% remission rate in acute leukemia ^53, 54^. However, one caveat to CARs is that these cells are long-lived and can have substantial off-target effects.

## Silence

The success of HAART has demonstrated that keeping the virus suppressed results in markedly healthier individuals. Resting infected cells do not produce HIV. Thus, these strategies rely on reducing T cell activation, which should also reduce the HIV-associated inflammation found in chronically infected individuals ^2^. JAK and STAT molecules are important signaling molecules associated with many cytokine receptors. Ruxolitinib and tofacitinib, two JAK inhibitors that are approved for the treatment of rheumatoid arthritis and myelofibrosis, were tested against HIV, HIV2, and simian HIV (SHIV). They inhibited HIV reactivation ^55^, and, furthermore, ruxolitinib attenuated encephalitis symptoms in infected humanized mice ^56^. Cyclosporine A, an immunosuppressant used primarily to prevent transplant rejection ^57^, inhibits T cell proliferation by blocking IL-2 signaling in T cells ^58^. Infected patients treated with cyclosporine A had some T cell recovery ^59^ but limited suppression of HIV replication ^60, 61^.

The inhibitor didehydro-cortistatin A (dCA) acts via a suppressive mechanism that primarily targets HIV transcription. dCA binds to the basic domain in the HIV regulatory protein Tat, inhibits its interactions with the RNA response element TAR, and prevents its activation of HIV transcription ^62^. dCA inhibits HIV reactivation in cell lines, primary cells, and peripheral blood mononuclear cells (PBMCs) from HAART-suppressed patients ^62^. Furthermore, dCA may also contribute to continued HIV suppression by inhibiting inflammatory cytokine expression ^63^.

## Gene therapy

Recently, a number of groups have taken advantage of cutting edge gene therapy approaches to HIV cure. However, as with any gene therapy approach, the barriers include delivery, specificity, off-target effects, costs, and ethical concerns.

The single case of successful HIV cure was achieved by the reconstitution of the patient’s immune system with donor bone marrow containing a natural mutation in the CCR5 HIV co-receptor ^64^. This patient was treated for acute leukemia with several courses of total lymphoid irradiation followed by two separate bone marrow transplantations. Attempts to replicate this therapy used the Zn++ finger nuclease ^65^ and more recently CRISPR/Cas9 targeting of CCR5 to induce the delta 32 mutation in patients’ own hematopoietic cells ^66, 67^, which were then returned to the host. Since only mature cells were used, the effects of these manipulated cells were not permanent ^65^. Recent work using CRISPR/Cas9 to target the second HIV co-receptor, CXCR4, has also yielded promising results ^68, 69^.

While HIV and SIV are highly related viruses, HIV cannot infect non-human primates, as their restriction factors block HIV infection more effectively than their human counterparts ^70^. Therefore, altering human restriction factors to behave like their simian counterparts represents an attractive strategy. One such factor is TRIM5. Of special interest is TRIM5 from owl monkeys, which is linked in frame to cyclophilin A, and this fusion protein blocks HIV ^71^. Using lentiviral vectors to deliver Trim-Cyp has blocked HIV effectively in cell lines and primary T cells ^72^. Additionally, it has been used successfully in a triple combination anti-HIV lentiviral vector approach in an infected humanized mouse model ^73^.

Recently, CRISPR/Cas9 technology has emerged as the most versatile and effective gene therapy approach. Using a DNA targeting strategy utilized by bacterial CRISPR, any number of specific guide RNAs can be loaded into the Cas9 protein to target specific areas of DNA for knock out or knock in of genes ^74^. Similarly, this technology has been used to knock out and reactivate latent HIV. Targeting various regions of the HIV LTR inactivated the virus in infected cell lines ^75^ and prevented their reinfection ^76^. However, viral target sequences can mutate, and HIV LTR-specific guide RNA can fail to recognize and target the mutant sequences, preventing long-term eradication by this method ^77^. To reactivate HIV, a defective Cas9 protein (dCas9) is used, which is fused to four copies of the herpes simplex VP16 activation domain (VP64) or a synergistic activation mediator (SAM) complex. Again, guide RNAs bring these dCas9 activators to the initiated transcription machinery. This targeting results in potent reactivation in latently infected cell lines ^78– 80^.

## Summary

Although HIV infection in the era of HAART has become a manageable chronic infection, problems with adherence to drug regiment, co-morbidities, and the emergence of drug resistance emphasize the need for continued research into HIV cure. Since the barrier to cure is the HIV reservoir, targeting this persistent virus is critical. The approaches detailed in this review represent a spectrum of the current research: however, eliminating the remaining 10 ^6^ to 10 ^8^ latently infected cells ^81^ will require a combination of approaches. Mechanisms, such as HIV reactivation, will reveal hidden virus. However, the severely crippled immune system and further decreased CTL function indicate that it must be paired with the boosting of anti-viral host defenses. Likewise, keeping latent HIV in a suppressed state could keep HIV+ patients relatively healthy but less able to resist other infections and/or cancer. Using gene therapy to create a parallel immune system, where cells resist HIV infection, could complement all other approaches but is not scalable or affordable in resource-poor countries. While none of these approaches represent the eradication of HIV, combining several treatment modalities could bring us closer to a functional cure, where prolonged HAART-free and disease-free intervals would be achieved in infected patients.

## References

[1] UNAIDS: AIDS by the numbers 2015. Joint United Nations Programme on HIV/AIDS (UNAIDS).2015 Reference Source

[2] Marin B Thiébaut R Bucher HC: Non-AIDS-defining deaths and immunodeficiency in the era of combination antiretroviral therapy. *AIDS.* 2009;23(13):1743–53. 10.1097/QAD.0b013e32832e9b78 19571723 PMC3305466

[3] Buzón MJ Massanella M Llibre JM: HIV-1 replication and immune dynamics are affected by raltegravir intensification of HAART-suppressed subjects. *Nat Med.* 2010;16(4):460–5. 10.1038/nm.2111 20228817

[4] Lorenzo-Redondo R Fryer HR Bedford T: Persistent HIV-1 replication maintains the tissue reservoir during therapy. *Nature.* 2016;530(7588):51–6. 10.1038/nature16933 26814962 PMC4865637

[5] Chun TW Stuyver L Mizell SB: Presence of an inducible HIV-1 latent reservoir during highly active antiretroviral therapy. *Proc Natl Acad Sci U S A.* 1997;94(24):13193–7. 10.1073/pnas.94.24.13193 9371822 PMC24285

[6] Finzi D Hermankova M Pierson T: Identification of a reservoir for HIV-1 in patients on highly active antiretroviral therapy. *Science.* 1997;278(5341):1295–300. 10.1126/science.278.5341.1295 9360927

[7] Wong JK Hezareh M Günthard HF: Recovery of replication-competent HIV despite prolonged suppression of plasma viremia. *Science.* 1997;278(5341):1291–5. 10.1126/science.278.5341.1291 9360926

[8] Day CL Kaufmann DE Kiepiela P: PD-1 expression on HIV-specific T cells is associated with T-cell exhaustion and disease progression. *Nature.* 2006;443(7109):350–4. 10.1038/nature05115 16921384

[9] Kaufmann DE Kavanagh DG Pereyra F: Upregulation of CTLA-4 by HIV-specific CD4 ^+^ T cells correlates with disease progression and defines a reversible immune dysfunction. *Nat Immunol.* 2007;8(11):1246–54. 10.1038/ni1515 17906628

[10] Callahan MK Wolchok JD: At the bedside: CTLA-4- and PD-1-blocking antibodies in cancer immunotherapy. *J Leukoc Biol.* 2013;94(1):41–53. 10.1189/jlb.1212631 23667165 PMC4051187

[11] Jones RB Walker BD: HIV-specific CD8 ^+^ T cells and HIV eradication. *J Clin Invest.* 2016;126(2):455–63. 10.1172/JCI80566 26731469 PMC4731167

[12] Renga B Francisci D D'Amore C: HIV-1 infection is associated with changes in nuclear receptor transcriptome, pro-inflammatory and lipid profile of monocytes. *BMC Infect Dis.* 2012;12:274. 10.1186/1471-2334-12-274 23106848 PMC3528633

[13] Yadav A Collman RG: CNS inflammation and macrophage/microglial biology associated with HIV-1 infection. *J Neuroimmune Pharmacol.* 2009;4(4):430–47. 10.1007/s11481-009-9174-2 19768553 PMC5935112

[14] Brooks DG Ha SJ Elsaesser H: IL-10 and PD-L1 operate through distinct pathways to suppress T-cell activity during persistent viral infection. *Proc Natl Acad Sci U S A.* 2008;105(51):20428–33. 10.1073/pnas.0811139106 19075244 PMC2629263

[15] Rigopoulou EI Abbott WG Haigh P: Blocking of interleukin-10 receptor--a novel approach to stimulate T-helper cell type 1 responses to hepatitis C virus. *Clin Immunol.* 2005;117(1):57–64. 10.1016/j.clim.2005.06.003 16006191

[16] Levy Y Lacabaratz C Weiss L: Enhanced T cell recovery in HIV-1-infected adults through IL-7 treatment. *J Clin Invest.* 2009;119(4):997–1007. 10.1172/JCI38052 19287090 PMC2662568

[17] Scripture-Adams DD Brooks DG Korin YD: Interleukin-7 induces expression of latent human immunodeficiency virus type 1 with minimal effects on T-cell phenotype. *J Virol.* 2002;76(24):13077–82. 10.1128/JVI.76.24.13077-13082.2002 12438635 PMC136703

[18] Lévy Y Sereti I Tambussi G: Effects of recombinant human interleukin 7 on T-cell recovery and thymic output in HIV-infected patients receiving antiretroviral therapy: results of a phase I/IIa randomized, placebo-controlled, multicenter study. *Clin Infect Dis.* 2012;55(2):291–300. 10.1093/cid/cis383 22550117 PMC3381639

[19] Seay K Church C Zheng JH: *In Vivo* Activation of Human NK Cells by Treatment with an Interleukin-15 Superagonist Potently Inhibits Acute *In Vivo* HIV-1 Infection in Humanized Mice. *J Virol.* 2015;89(12):6264–74. 10.1128/JVI.00563-15 25833053 PMC4474292

[20] Bartholomeeusen K Xiang Y Fujinaga K: Bromodomain and extra-terminal (BET) bromodomain inhibition activate transcription via transient release of positive transcription elongation factor b (P-TEFb) from 7SK small nuclear ribonucleoprotein. *J Biol Chem.* 2012;287(43):36609–16. 10.1074/jbc.M112.410746 22952229 PMC3476326

[21] Yu W Ramakrishnan R Wang Y: Cyclin T1-dependent genes in activated CD4 ^+^ T and macrophage cell lines appear enriched in HIV-1 co-factors. *PLoS One.* 2008;3(9):e3146. 10.1371/journal.pone.0003146 18773076 PMC2519787

[22] Bartholomeeusen K Fujinaga K Xiang Y: Histone deacetylase inhibitors (HDACis) that release the positive transcription elongation factor b (P-TEFb) from its inhibitory complex also activate HIV transcription. *J Biol Chem.* 2013;288(20):14400–7. 10.1074/jbc.M113.464834 23539624 PMC3656295

[23] Rasmussen TA Schmeltz Søgaard O Brinkmann C: Comparison of HDAC inhibitors in clinical development: effect on HIV production in latently infected cells and T-cell activation. *Hum Vaccin Immunother.* 2013;9(5):993–1001. 10.4161/hv.23800 23370291 PMC3899169

[24] Wei DG Chiang V Fyne E: Histone deacetylase inhibitor romidepsin induces HIV expression in CD4 T cells from patients on suppressive antiretroviral therapy at concentrations achieved by clinical dosing. *PLoS Pathog.* 2014;10(4):e1004071. 10.1371/journal.ppat.1004071 24722454 PMC3983056

[25] Contreras X Schweneker M Chen CS: Suberoylanilide hydroxamic acid reactivates HIV from latently infected cells. *J Biol Chem.* 2009;284(11):6782–9. 10.1074/jbc.M807898200 19136668 PMC2652322

[26] Routy JP Tremblay CL Angel JB: Valproic acid in association with highly active antiretroviral therapy for reducing systemic HIV-1 reservoirs: results from a multicentre randomized clinical study. *HIV Med.* 2012;13(5):291–6. 10.1111/j.1468-1293.2011.00975.x 22276680

[27] Boehm D Calvanese V Dar RD: BET bromodomain-targeting compounds reactivate HIV from latency via a Tat-independent mechanism. *Cell Cycle.* 2013;12(3):452–62. 10.4161/cc.23309 23255218 PMC3587446

[28] Spina CA Anderson J Archin NM: An in-depth comparison of latent HIV-1 reactivation in multiple cell model systems and resting CD4+ T cells from aviremic patients. *PLoS Pathog.* 2013;9(12):e1003834. 10.1371/journal.ppat.1003834 24385908 PMC3873446

[29] Blazkova J Chun TW Belay BW: Effect of histone deacetylase inhibitors on HIV production in latently infected, resting CD4 ^+^ T cells from infected individuals receiving effective antiretroviral therapy. *J Infect Dis.* 2012;206(5):765–9. 10.1093/infdis/jis412 22732922 PMC3491743

[30] Archin NM Liberty AL Kashuba AD: Administration of vorinostat disrupts HIV-1 latency in patients on antiretroviral therapy. *Nature.* 2012;487(7408):482–5. 10.1038/nature11286 22837004 PMC3704185

[31] Korin YD Brooks DG Brown S: Effects of prostratin on T-cell activation and human immunodeficiency virus latency. *J Virol.* 2002;76(16):8118–23. 10.1128/JVI.76.16.8118-8123.2002 12134017 PMC155166

[32] Pérez M de Vinuesa AG Sanchez-Duffhues G: Bryostatin-1 synergizes with histone deacetylase inhibitors to reactivate HIV-1 from latency. *Curr HIV Res.* 2010;8(6):418–29. 10.2174/157016210793499312 20636281

[33] Hori T Barnor J Huu TN: Procyanidin trimer C1 derived from *Theobroma cacao* reactivates latent human immunodeficiency virus type 1 provirus. *Biochem Biophys Res Commun.* 2015;459(2):288–93. 10.1016/j.bbrc.2015.02.102 25727021

[34] Wang C Yang S Lu H: A Natural Product from *Polygonum cuspidatum* Sieb. Et Zucc. Promotes Tat-Dependent HIV Latency Reversal through Triggering P-TEFb's Release from 7SK snRNP. *PLoS One.* 2015;10(11):e0142739. 10.1371/journal.pone.0142739 26569506 PMC4646521

[35] Beans EJ Fournogerakis D Gauntlett C: Highly potent, synthetically accessible prostratin analogs induce latent HIV expression *in vitro* and *ex vivo*. *Proc Natl Acad Sci U S A.* 2013;110(29):11698–703. 10.1073/pnas.1302634110 23812750 PMC3718093

[36] DeChristopher BA Loy BA Marsden MD: Designed, synthetically accessible bryostatin analogues potently induce activation of latent HIV reservoirs *in vitro*. *Nat Chem.* 2012;4(9):705–10. 10.1038/nchem.1395 22914190 PMC3428736

[37] Wender PA Nakagawa Y Near KE: Computer-guided design, synthesis, and protein kinase C affinity of a new salicylate-based class of bryostatin analogs. *Org Lett.* 2014;16(19):5136–9. 10.1021/ol502491f 25238583 PMC4334246

[38] Pandeló José D Bartholomeeusen K da Cunha RD: Reactivation of latent HIV-1 by new semi-synthetic ingenol esters. *Virology.* 2014;462-463:328–39. 10.1016/j.virol.2014.05.033 25014309 PMC4383768

[39] Jiang G Mendes EA Kaiser P: Reactivation of HIV latency by a newly modified Ingenol derivative via protein kinase Cδ-NF-κB signaling. *AIDS.* 2014;28(11):1555–66. 10.1097/QAD.0000000000000289 24804860 PMC4922310

[40] Abreu CM Price SL Shirk EN: Dual role of novel ingenol derivatives from *Euphorbia tirucalli* in HIV replication: inhibition of *de novo* infection and activation of viral LTR. *PLoS One.* 2014;9(5):e97257. 10.1371/journal.pone.0097257 24827152 PMC4020785

[41] Jiang G Mendes EA Kaiser P: Synergistic Reactivation of Latent HIV Expression by Ingenol-3-Angelate, PEP005, Targeted NF-kB Signaling in Combination with JQ1 Induced p-TEFb Activation. *PLoS Pathog.* 2015;11(7):e1005066. 10.1371/journal.ppat.1005066 26225771 PMC4520526

[42] Darcis G Kula A Bouchat S: An In-Depth Comparison of Latency-Reversing Agent Combinations in Various *In Vitro* and *Ex Vivo* HIV-1 Latency Models Identified Bryostatin-1+JQ1 and Ingenol-B+JQ1 to Potently Reactivate Viral Gene Expression. *PLoS Pathog.* 2015;11(7):e1005063. 10.1371/journal.ppat.1005063 26225566 PMC4520688

[43] Laird GM Bullen CK Rosenbloom DI: *Ex vivo* analysis identifies effective HIV-1 latency-reversing drug combinations. *J Clin Invest.* 2015;125(5):1901–12. 10.1172/JCI80142 25822022 PMC4463209

[44] Scully E Alter G: NK Cells in HIV Disease. *Curr HIV/AIDS Rep.* 2016;13(2):85–94. 10.1007/s11904-016-0310-3 27002078 PMC4821863

[45] Jones RB O'Connor R Mueller S: Histone deacetylase inhibitors impair the elimination of HIV-infected cells by cytotoxic T-lymphocytes. *PLoS Pathog.* 2014;10(8):e1004287. 10.1371/journal.ppat.1004287 25122219 PMC4133386

[46] Cicin-Sain L Sylwester AW Hagen SI: Cytomegalovirus-specific T cell immunity is maintained in immunosenescent rhesus macaques. *J Immunol.* 2011;187(4):1722–32. 10.4049/jimmunol.1100560 21765018 PMC3151292

[47] Hansen SG Ford JC Lewis MS: Profound early control of highly pathogenic SIV by an effector memory T-cell vaccine. *Nature.* 2011;473(7348):523–7. 10.1038/nature10003 21562493 PMC3102768

[48] Hansen SG Sacha JB Hughes CM: Cytomegalovirus vectors violate CD8 ^+^ T cell epitope recognition paradigms. *Science.* 2013;340(6135): 1237874. 10.1126/science.1237874 23704576 PMC3816976

[49] Halper-Stromberg A Nussenzweig MC: Towards HIV-1 remission: potential roles for broadly neutralizing antibodies. *J Clin Invest.* 2016;126(2):415–23. 10.1172/JCI80561 26752643 PMC4731188

[50] Chun TW Murray D Justement JS: Broadly neutralizing antibodies suppress HIV in the persistent viral reservoir. *Proc Natl Acad Sci U S A.* 2014;111(36):13151–6. 10.1073/pnas.1414148111 25157148 PMC4246957

[51] Halper-Stromberg A Lu CL Klein F: Broadly neutralizing antibodies and viral inducers decrease rebound from HIV-1 latent reservoirs in humanized mice. *Cell.* 2014;158(5):989–99. 10.1016/j.cell.2014.07.043 25131989 PMC4163911

[52] Bruel T Guivel-Benhassine F Amraoui S: Elimination of HIV-1-infected cells by broadly neutralizing antibodies. *Nat Commun.* 2016;7: 10844. 10.1038/ncomms10844 26936020 PMC4782064

[53] Grupp SA Kalos M Barrett D: Chimeric antigen receptor-modified T cells for acute lymphoid leukemia. *N Engl J Med.* 2013;368(16):1509–18. 10.1056/NEJMoa1215134 23527958 PMC4058440

[54] Maude SL Frey N Shaw PA: Chimeric antigen receptor T cells for sustained remissions in leukemia. *N Engl J Med.* 2014;371(16):1507–17. 10.1056/NEJMoa1407222 25317870 PMC4267531

[55] Gavegnano C Detorio M Montero C: Ruxolitinib and tofacitinib are potent and selective inhibitors of HIV-1 replication and virus reactivation *in vitro*. *Antimicrob Agents Chemother.* 2014;58(4):1977–86. 10.1128/AAC.02496-13 24419350 PMC4023721

[56] Haile WB Gavegnano C Tao S: The Janus kinase inhibitor ruxolitinib reduces HIV replication in human macrophages and ameliorates HIV encephalitis in a murine model. *Neurobiol Dis.* 2016; pii: S0969-9961(16)30028-6. 10.1016/j.nbd.2016.02.007 26851503 PMC4907871

[57] Starzl TE Weil R 3rd Iwatsuki S: The use of cyclosporin A and prednisone in cadaver kidney transplantation. *Surg Gynecol Obstet.* 1980;151(1):17–26. 6992310 PMC2727074

[58] Bunjes D Hardt C Röllinghoff M: Cyclosporin A mediates immunosuppression of primary cytotoxic T cell responses by impairing the release of interleukin 1 and interleukin 2. *Eur J Immunol.* 1981;11(8):657–61. 10.1002/eji.1830110812 6456149

[59] Andrieu JM Even P Venet A: Effects of cyclosporin on T-cell subsets in human immunodeficiency virus disease. *Clin Immunol Immunopathol.* 1988;47(2):181–98. 10.1016/0090-1229(88)90071-2 3258211

[60] Markowitz M Vaida F Hare CB: The virologic and immunologic effects of cyclosporine as an adjunct to antiretroviral therapy in patients treated during acute and early HIV-1 infection. *J Infect Dis.* 2010;201(9):1298–302. 10.1086/651664 20235838 PMC2851487

[61] Rizzardi GP Harari A Capiluppi B: Treatment of primary HIV-1 infection with cyclosporin A coupled with highly active antiretroviral therapy. *J Clin Invest.* 2002;109(5):681–8. 10.1172/JCI14522 11877476 PMC150896

[62] Mousseau G Kessing CF Fromentin R: The Tat Inhibitor Didehydro-Cortistatin A Prevents HIV-1 Reactivation from Latency. *MBio.* 2015;6(4):e00465. 10.1128/mBio.00465-15 26152583 PMC4495168

[63] Mediouni S Jablonski J Paris JJ: Didehydro-cortistatin A inhibits HIV-1 Tat mediated neuroinflammation and prevents potentiation of cocaine reward in Tat transgenic mice. *Curr HIV Res.* 2015;13(1):64–79. 10.2174/1570162X13666150121111548 25613133 PMC4416414

[64] Hütter G Nowak D Mossner M: Long-term control of HIV by *CCR5* Delta32/Delta32 stem-cell transplantation. *N Engl J Med.* 2009;360(7):692–8. 10.1056/NEJMoa0802905 19213682

[65] Tebas P Stein D Tang WW: Gene editing of *CCR5* in autologous CD4 T cells of persons infected with HIV. *N Engl J Med.* 2014;370(10):901–10. 10.1056/NEJMoa1300662 24597865 PMC4084652

[66] Ye L Wang J Beyer AI: Seamless modification of wild-type induced pluripotent stem cells to the natural CCR5Δ32 mutation confers resistance to HIV infection. *Proc Natl Acad Sci U S A.* 2014;111(26):9591–6. 10.1073/pnas.1407473111 24927590 PMC4084478

[67] Wang W Ye C Liu J: *CCR5* gene disruption via lentiviral vectors expressing Cas9 and single guided RNA renders cells resistant to HIV-1 infection. *PLoS One.* 2014;9(12):e115987. 10.1371/journal.pone.0115987 25541967 PMC4277423

[68] Schumann K Lin S Boyer E: Generation of knock-in primary human T cells using Cas9 ribonucleoproteins. *Proc Natl Acad Sci U S A.* 2015;112(33):10437–42. 10.1073/pnas.1512503112 26216948 PMC4547290

[69] Hou P Chen S Wang S: Genome editing of *CXCR4* by CRISPR/cas9 confers cells resistant to HIV-1 infection. *Sci Rep.* 2015;5: 15577. 10.1038/srep15577 26481100 PMC4612538

[70] Chan E Towers GJ Qasim W: Gene therapy strategies to exploit TRIM derived restriction factors against HIV-1. *Viruses.* 2014;6(1):243–63. 10.3390/v6010243 24424502 PMC3917441

[71] Carthagena L Parise MC Ringeard M: Implication of TRIM alpha and TRIMCyp in interferon-induced anti-retroviral restriction activities. *Retrovirology.* 2008;5:59. 10.1186/1742-4690-5-59 18613956 PMC2483995

[72] Neagu MR Ziegler P Pertel T: Potent inhibition of HIV-1 by TRIM5-cyclophilin fusion proteins engineered from human components. *J Clin Invest.* 2009;119(10):3035–47. 10.1172/JCI39354 19741300 PMC2752079

[73] Walker JE Chen RX McGee J: Generation of an HIV-1-resistant immune system with CD34 ^+^ hematopoietic stem cells transduced with a triple-combination anti-HIV lentiviral vector. *J Virol.* 2012;86(10):5719–29. 10.1128/JVI.06300-11 22398281 PMC3347262

[74] Wright AV Nuñez JK Doudna JA: Biology and Applications of CRISPR Systems: Harnessing Nature's Toolbox for Genome Engineering. *Cell.* 2016;164(1–2):29–44. 10.1016/j.cell.2015.12.035 26771484

[75] Ebina H Misawa N Kanemura Y: Harnessing the CRISPR/Cas9 system to disrupt latent HIV-1 provirus. *Sci Rep.* 2013;3: 2510. 10.1038/srep02510 23974631 PMC3752613

[76] Hu W Kaminski R Yang F: RNA-directed gene editing specifically eradicates latent and prevents new HIV-1 infection. *Proc Natl Acad Sci U S A.* 2014;111(31):11461–6. 10.1073/pnas.1405186111 25049410 PMC4128125

[77] Wang G Zhao N Berkhout B: CRISPR-Cas9 Can Inhibit HIV-1 Replication but NHEJ Repair Facilitates Virus Escape. *Mol Ther.* 2016;24(3):522–6. 10.1038/mt.2016.24 26796669 PMC4786927

[78] Zhang Y Yin C Zhang T: CRISPR/gRNA-directed synergistic activation mediator (SAM) induces specific, persistent and robust reactivation of the HIV-1 latent reservoirs. *Sci Rep.* 2015;5: 16277. 10.1038/srep16277 26538064 PMC4633726

[79] Saayman SM Lazar DC Scott TA: Potent and Targeted Activation of Latent HIV-1 Using the CRISPR/dCas9 Activator Complex. *Mol Ther.* 2016;24(3):488–98. 10.1038/mt.2015.202 26581162 PMC4786915

[80] Limsirichai P Gaj T Schaffer DV: CRISPR-mediated Activation of Latent HIV-1 Expression. *Mol Ther.* 2016;24(3):499–507. 10.1038/mt.2015.213 26607397 PMC4786916

[81] Massanella M Richman DD: Measuring the latent reservoir *in vivo*. *J Clin Invest.* 2016;126(2):464–72. 10.1172/JCI80567 26829625 PMC4731179

